# Random forests algorithm boosts genetic risk prediction of systemic lupus erythematosus

**DOI:** 10.3389/fgene.2022.902793

**Published:** 2022-08-15

**Authors:** Wen Ma, Yu-Lung Lau, Wanling Yang, Yong-Fei Wang

**Affiliations:** ^1^ Department of Paediatrics and Adolescent Medicine, The University of Hong Kong, Hong Kong, China; ^2^ Shenzhen Futian Hospital for Rheumatic Diseases, Shenzhen, China

**Keywords:** systemic lupus erythematosus (SLE), SLE early detection, polygenic risk score, machine learning, random forests

## Abstract

Patients with systemic lupus erythematosus (SLE) present varied clinical manifestations, posing a diagnostic challenge for physicians. Genetic factors substantially contribute to SLE development. A polygenic risk scoring (PRS) model has been used to estimate the genetic risk of SLE in individuals. However, this approach assumes independent and additive contribution of genetic variants to disease development. We aimed to improve the accuracy of SLE prediction using machine-learning algorithms. We applied random forest (RF), support vector machine (SVM), and artificial neural network (ANN) to classify SLE cases and controls using the data from our previous genome-wide association studies (GWAS) conducted in either Chinese or European populations, including a total of 19,208 participants. The overall performances of these predictors were assessed by the value of area under the receiver-operator curve (AUC). The analyses in the Chinese GWAS showed that the RF model significantly outperformed other predictors, achieving a mean AUC value of 0.84, a 13% improvement upon the PRS model (AUC = 0.74). At the optimal cut-off, the RF predictor reached a sensitivity of 84% with a specificity of 68% in SLE classification. To validate these results, similar analyses were repeated in the European GWAS, and the RF model consistently outperformed other algorithms. Our study suggests that the RF model could be an additional and powerful predictor for SLE early diagnosis.

## Introduction

Systemic lupus erythematosus (SLE) is a complex autoimmune disease that affects multiple organ systems. SLE patients often show varied clinical manifestations, ranging from mild skin lesion to lethal renal involvement. The clinical heterogeneity of SLE poses a diagnostic challenge for physicians. Recent studies have shown that it usually takes 4–6 years for SLE patients to be correctly diagnosed from the time the first symptoms start to appear and more than 60% of the patients were misdiagnosed before receiving a comprehensive examination (Al Sawah et al., 2015). Primary care physicians (PCPs) play a central role in early diagnosis. However, a previous study showed that only 56% of SLE patients diagnosed by PCPs met one of three major criteria for SLE classification (Lawrence et al., 1987). Delayed diagnosis and treatment often result in more severe disease outcomes, increasing the chance of irreversible organ damages (Kernder et al., 2021). Early detection and intervention are essential for achieving optimal treatment outcomes for patients.

Genetic assessment may become an additional tool for early diagnosis of SLE as genetic factors explain about 43%–66% of SLE development (Lawrence et al., 1987; Wang et al., 2007; Kuo et al., 2015). Polygenic risk scoring (PRS) methods have been applied in SLE prediction and stratification (Reid et al., 2019; Chen et al., 2020; Wang et al., 2021; Wang et al., 2022). However, the classic PRS method simply aggregates the number of risk alleles for a subset of linkage disequilibrium (LD)-independent variants that exceed an association *p*-value threshold, and the contribution of each variant is usually weighted by the effect-size estimated in the relevant genome-wide association studies (GWASs) (Choi et al., 2020). This procedure will select the variants with inflated effects due to the influence of Winner’s curse (Shi et al., 2016), and the hard filtering will remove multiple variants with small but non-negligible effects. Recently, a range of modified PRS methods have been developed (Pain et al., 2021). These methods apply shrinkage methods to reduce overfitting of genetic effects caused by Winner’s curse and incorporate genome-wide variants into a model to maximize the signals captured. Our recent study showed that the lassosum-based PRS model (Mak et al., 2017) achieved an overall performance with the area under the receiver-operator curve (AUC) value of 0.76 for SLE prediction in Chinese population (Wang et al., 2021).

Despite a great improvement of novel PRS methods, the assumption of independent and additive contribution of genetic variants to disease development hinders their further improvement. Recent studies have shown that non-additive and epistasis effects could explain a substantial proportion of heritability for complex diseases (Wei et al., 2014; Guindo-Martinez et al., 2021). Compared to the PRS model, supervised machine learning (ML) algorithms, using multivariate and non-parametric methods, may have stronger power to capture signals from non-linearly and non-normally distributed data (Ho et al., 2019). Here, we compare the performances of three widely used ML algorithms, namely, random forest (RF), support vector machine (SVM), and artificial neural network (ANN) with the lassosum-based PRS model in predicting SLE development using the data from our previous GWAS that were conducted in either Chinese (3,720 cases and 5,303 controls) or European populations (3,670 cases and 6,515 controls).

## Methods

### Data source

The individuals with raw genotype data were retrieved from our previous SLE GWAS (Song et al., 2021; Wang et al., 2021) (Supplementary Table S1), including a total of 9,023 individuals with Chinese ancestry (3,720 SLE cases and 5,303 controls) and 10,185 individuals with European ancestry (3,670 cases and 6,515 controls). All the patients fulfilled the revised criteria of the American College of Rheumatology for diagnosis of SLE (ACR-97) (Hochberg, 1997). Quality control and imputation analyses for individual-level genotype data were performed as described in our previous studies (Wang et al., 2018; Wang et al., 2021; Wang et al., 2022). To obtain a set of common and well-imputed variants for subsequent analyses, variants who met the following criteria were included: 1) minor allele frequency greater than 1%; 2) imputation INFO scores greater than 0.90; 3) passing the test of Hardy–Weinberg equilibrium (*p*-value >1E−04); 4) variants in the HapMap 3 reference panel. A total of 958,649 and 923,949 variants in autosomes met these criteria in the Chinese and European datasets, respectively. These variants were used for subsequent analyses.

### Polygenic risk score calculation

The polygenic scores for individuals were calculated using the lassosum model (Mak et al., 2017), which has been proven to be one of the best PRS models (Pain et al., 2021). The GWAS association summary statistics were calculated using the logistic model in PLINK, and the first three genetic principal components and batch effects were controlled in the association analyses. All variants that met the selection criteria were applied to the lassosum-based PRS model for SLE prediction in either Chinese or European datasets. The parameters of “s” and “lambda” in the model were further tuned using the raw genotyped data of training samples.

### Supervised machine learning classifiers

In this study, we constructed three ML models, random forest (RF), support vector machine (SVM) and artificial neural network (ANN), for SLE prediction. Considering that high-dimensional data could overwhelm computational resources and exacerbate the overfitting problem for these ML predictors, we performed a two-step SNP selection strategy to remove the variants that are in high linkage disequilibrium (LD). We first conducted the clumping function in PLINK to remove those highly correlated genetic variants (r^2^ > 0.3). After that, we performed lasso-logistic regression to further reduce the dimensionality using the “glmnet” function in the R package (Friedman et al., 2010). Though the two-step selection process, a total of 5,317 and 7,713 variants were included in the Chinese and European datasets, respectively. The numbers were below the sample size, and these variants were applied to the ML predictors.

The RF predictor was constructed using the function of RandomForestClassifier in the scikit-learn package (Pedregosa et al., 2011). The SVM predictor was constructed by using the function of sklearn.svm.SVC in the scikit-learn package. The ANN predictor was constructed by using the Keras, a Python library for developing deep learning models (Gulli and Pal, 2017). To tune the parameters for ML models, we used a subset of Chinese data that was collected from Hong Kong (HK; 1,604 cases and 3,324 controls) to train the models with varied settings of parameters and used the samples collected from Guangzhou (GZ; 1,604 cases and 985 controls) as a validation dataset.

For the RF model, we evaluated the impact of tree setting on the performance of SLE prediction. The HK data were used to train the RF model, with the number of trees ranging from 100 to 1,500. We observed that the AUC values for the validation dataset were positively associated with the number of trees used, but the performance nearly leveled off when the number was set to be 800 (Supplementary Figure S1A). Thus, eight hundred trees were set to construct the RF model for SLE prediction in the following study.

For the SVM model, we investigated the impact of kernel functions on the performance of SLE prediction. Three types of kernel functions, namely, radial basis function (“rbf”), polynomial (“poly”) and linear kernels were used to train the algorithm. The model trained by the “poly” function slightly outperformed other models (Supplementary Figure S1B). Thus, we selected the “poly” function to build the SVM model in the following study.

For the ANN model, the setting of hyperparameters is shown in Supplementary Table S2. We assessed the performance of the model with a depth of two hidden layers (512 and 256 nodes in respective layers) or three hidden layers (1,024, 512, and 256 nodes in respective layers). Meanwhile, we also evaluated the effect of varied learning rates (0.1, 0.01, and 0.001), which controls the magnitude of weight update and is the most important hyperparameter in the algorithm (Goodfellow et al., 2016). As shown in Supplementary Figure S1C, we observed that the model with a setting of three hidden layers and a learning rate of 0.001 (AUC = 0.626) performed slightly better than the model constructed by the three hidden layers but with a learning rate of 0.01 (AUC = 0.622). However, the small improvement was made at the expense of taking nearly an extra hour for training. Considering the balance between accuracy and resource usage, we constructed the ANN model with the setting of three hidden layers and a learning rate of 0.01 for SLE prediction in the following study.

### Evaluating performance of predictors

The overall performance for these predictors was assessed by the AUC value which is widely used to evaluate how well a predictor can identify the true state of subjects in a test. The value ranges from 0 to 1, and a higher value indicates a better performance in a diagnostic test. Sensitivity and specificity were also calculated given a specific cutoff to determine cases and controls.

## Results

**FIGURE 1:**
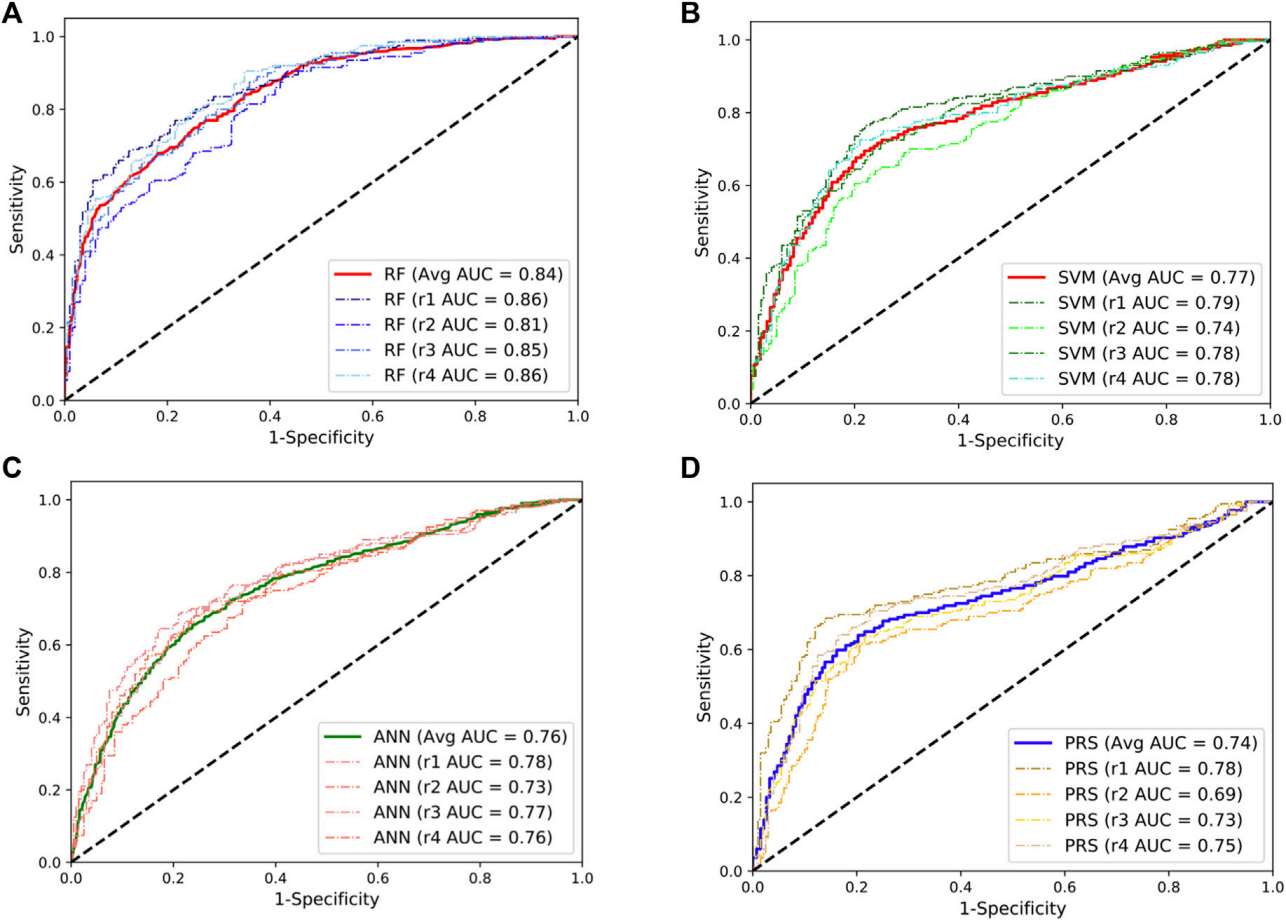
Performances of the random forests (RF; **(A)**), support vector machine (SVM; **(B)**), artificial neural network (ANN; **(C)**), and the lassosum-based polygenic risk scoring (PRS) model **(D)** in predicting the development of SLE in Chinese population. The dashed line indicates the performance for each repeat. The solid lines indicate the averaged performance among the four repeats.

We first evaluated the performance of three supervised ML predictors (RF, SVM, and ANN) and the lassosum-based PRS model using the SLE GWAS from Chinese populations (3720 cases and 5303 controls). To maximize the power of these predictors, we randomly selected 200 SLE cases and 200 controls from the GWAS as a testing dataset and trained the predictors using the remaining samples. This procedure was repeated four times to overcome estimation bias. The flowchart of data processes is shown in Supplementary Figure S2. The results showed that the RF model achieved a mean AUC of 0.84, significantly outperforming other predictors (Figure 1). The performance of SVM and ANN models was comparable, with the mean AUC value of 0.77 and 0.76, respectively, which was slightly higher than that of the PRS model (mean AUC = 0.74). In addition, we randomly split the data into two equal parts and used one half for training and the other half for testing. We observed similar results, and the RF predictor still outperformed other models for SLE prediction (Supplementary Figure S3).

Given the fact that the delayed treatment could result in irreversible organ damages, a more sensitive diagnostic test would be preferred for primary assessment. Following this line, we set a cut-off to classify cases and control at the point where the predictors can reach a sensitivity of 80% in the classification. The corresponding specificity at this point was 71%, 59%, 57%, and 43% for the RF, SVM, ANN, and PRS models, respectively. At the optimal cut-off where the sum of sensitivity and specificity was maximized, the RF model achieved a sensitivity of 84% and a specificity of 68% in predicting SLE development. In addition, the RF model consumes much less computational time than other models (Supplementary Figure S4).

**FIGURE 2:**
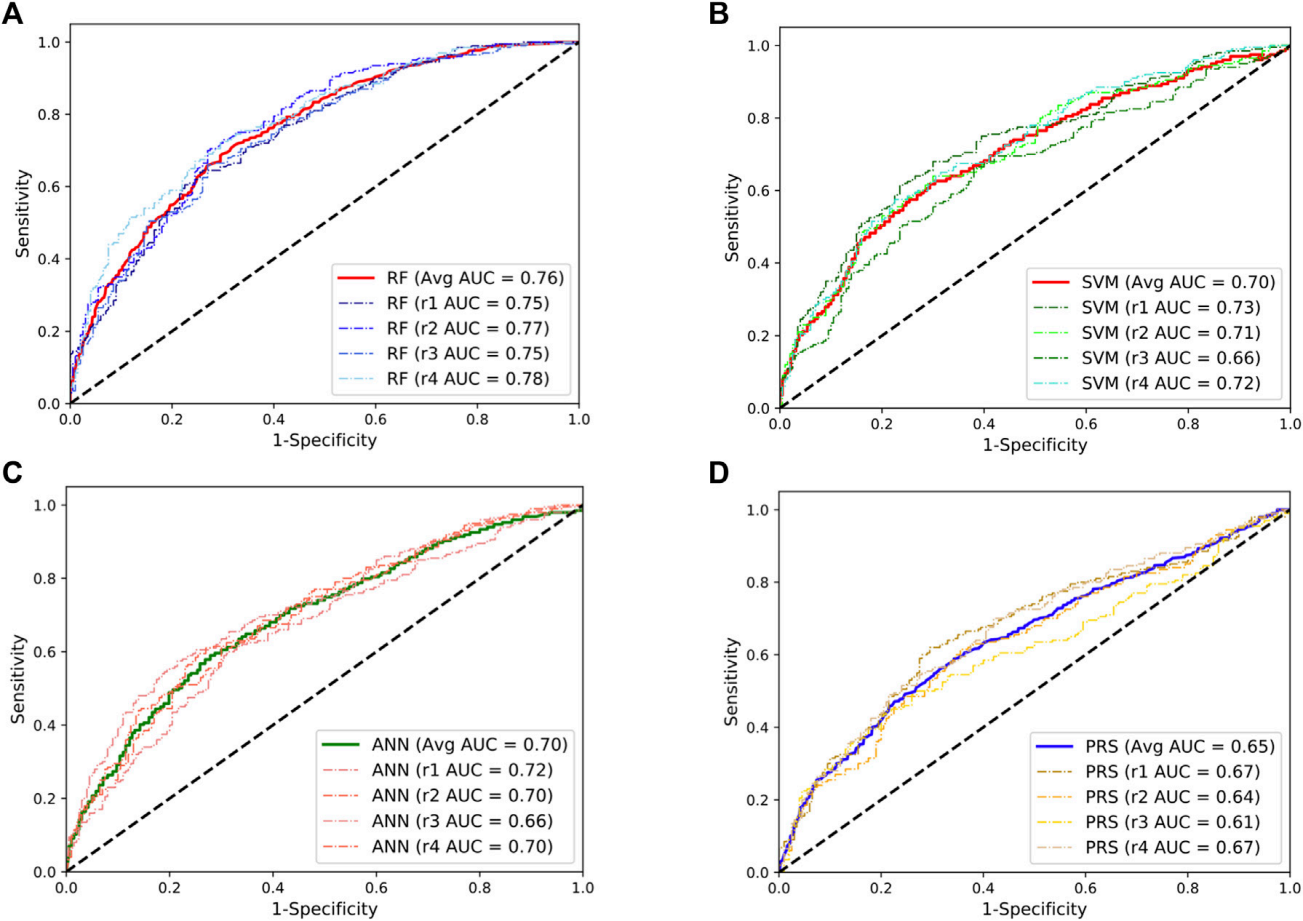
Performances of the random forests (RF; **(A)**), support vector machine (SVM; **(B)**), artificial neural network (ANN; **(C)**), and the lassosum-based polygenic risk scoring (PRS) model **(D)** in predicting the development of SLE in European population. The dashed line indicates the performance for each repeat. The solid lines indicate the averaged performance among the four repeats.

To validate these results, we repeated the abovementioned analyses using the SLE GWAS collected from European populations. We observed a similar pattern where the RF model achieved the best performance with a mean AUC value of 0.76, approximately a 17% improvement compared to the PRS model (Figure 2). Taken together, these results indicate that the RF model could be an additional and powerful tool for SLE classification and early detection.

## Discussion

The presence of autoantibodies, like antinuclear antibody (ANA) and double-stranded DNA (dsDNA), is one of the diagnostic criteria for SLE. However, these biomarkers are not specific for SLE (Fitch-Rogalsky et al., 2014), and the positive tests are likely present in the patients who are in the active phase of SLE. Genetic assessment could predict the disease risk long before disease onset as it relies on germline sequence that is stable throughout lifespan. With genome SNP genotyping or genome sequencing becoming more readily accessible, genetic assessment may greatly facilitate early diagnosis of SLE.

Our previous studies showed that the PRS model could achieve an AUC value of 0.76 in Chinese population (Wang et al., 2021). Here, we replicated the performance of the PRS model (mean AUC = 0.74) and demonstrated that the RF predictor improves the prediction of SLE development, achieving a mean AUC value of 0.84 in the Chinese dataset. In addition, we observed that the SVM and ANN predictors also slightly outperformed the PRS model, suggesting potential non-linear effects underlying the disease association. Previous studies also demonstrated that ML algorithms can boost predictive power of genetic assessments on inflammatory bowel disease (IBD) and celiac disease (Wei et al., 2013; Abraham et al., 2014). Taken together, these studies suggest an advancement of ML models in predicting the development of autoimmune diseases.

Unlike the PRS model, the ML models require genotype data for training, which may be limited by sample sizes that are available. However, the ML algorithms do not seem as sensitive to the change of sample size for training. We observed that the AUC value of the RF model was decreased from 0.84 when it was trained by nearly all available samples in the Chinese dataset (Figure 1A) to 0.82 when it was trained by one half of the data (Supplementary Figure S3A). However, more studies are needed to examine the effect of sample size on the performance of the model.

Understanding the relationships between subphenotypes of SLE and genetic risk may provide more insights into the clinical use of genetic findings. However, we have not curated the full clinical data in this study. It would be an intriguing question if clinical data are available as these ML algorithms can address multi-class problems and be applied to distinguish different subtypes of SLE. In summary, we recommend applying the RF model to estimate the genetic risk of SLE in individuals.

## Data Availability

The original contributions presented in the study are included in the article/Supplementary Material; further inquiries can be directed to the corresponding authors.
