# Supplementary material for: Random forests algorithm boosts genetic risk prediction of systemic lupus erythematosus
Source: Front Genet. 2022 Aug 15;13:902793. doi: 10.3389/fgene.2022.902793 (PMC9421562; doi:10.3389/fgene.2022.902793)
Supplement: Supplementary file 1 [file DataSheet1.PDF]

Supplementary figures and tables

Supplementary figure 1 Parameter optimization for the random forest, support vector machine and artificial neural network models for SLE prediction 2

Supplementary figure 2 An overview of workflow for the internal validation of predictive models based on the data from European and Chinese datasets. 3

Supplementary figure 3 Performance of the random forest, support vector machine, artificial neural network and the lassosum-based PRS models for SLE prediction...............4

Supplementary figure 4 Average CPU time cost for the random forest, support vector machine, artificial neural network and the PRS models 5

Supplementary table 1 Summary of SLE GWAS datasets in this study…………………..6

Supplementary table 2 setting of hyperparameters for the ANN model..................................7

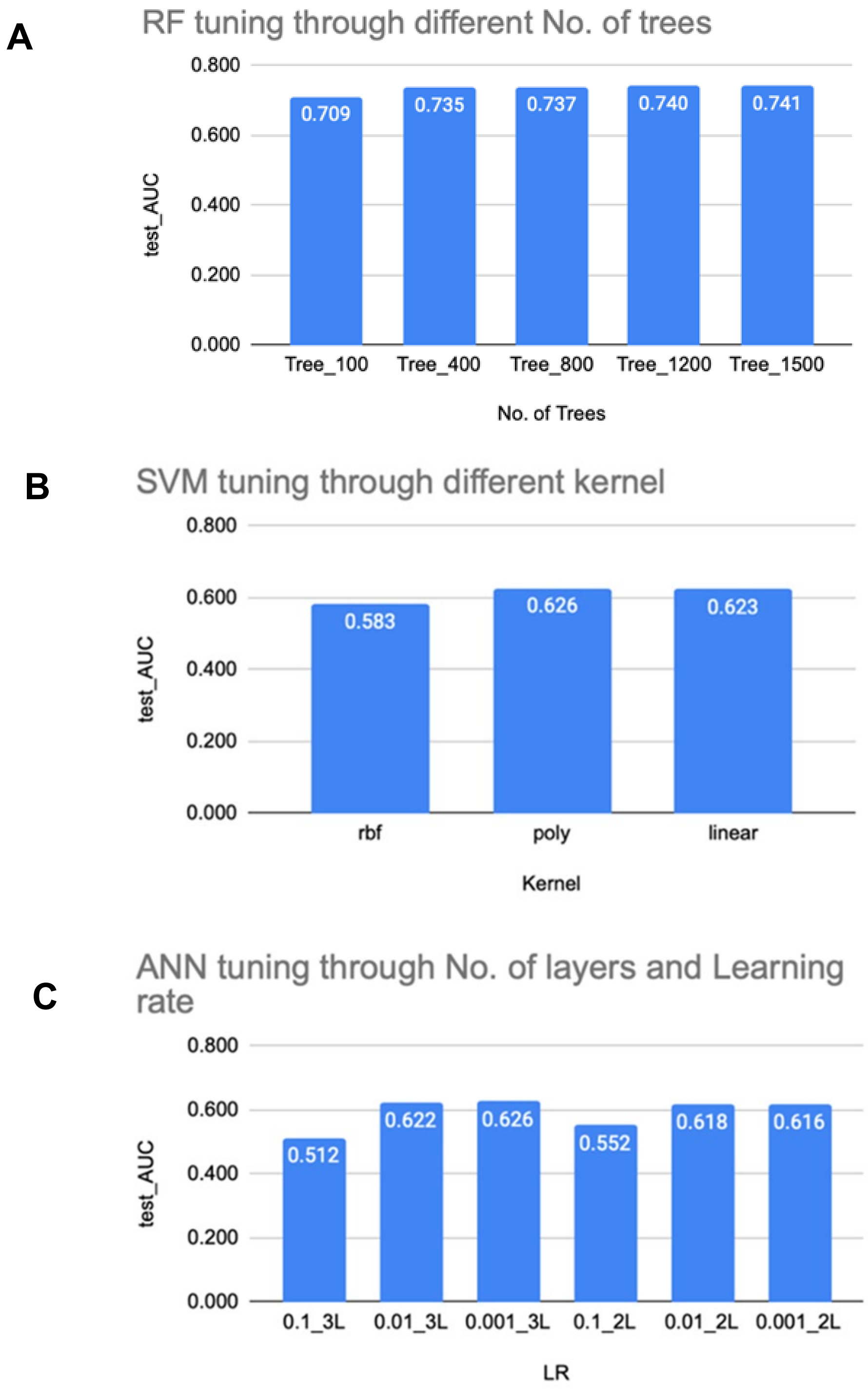

Supplementary figure 1 Parameter optimization for the random forest (RF, A), support vector machine (SVM, B) and artificial neural network (ANN, C) models for SLE prediction. Here, we used a subset of the Chinese GWAS that were collected from Hong Kong (HK; 1,604 cases and 3,324 controls) to train the models based on different settings of parameters, and used the samples collected from Guangzhou (GZ; 1,604 cases and 985 controls) as a validation dataset. For the ANN model, the value in front of "_" indicates the learning rate, and "3L" represents the models included three hidden layers with 1024, 512 and 256 nodes in respective layers, and "2L" indicates the models constructed by two hidden layers with 512 and 256 nodes in respective layers.

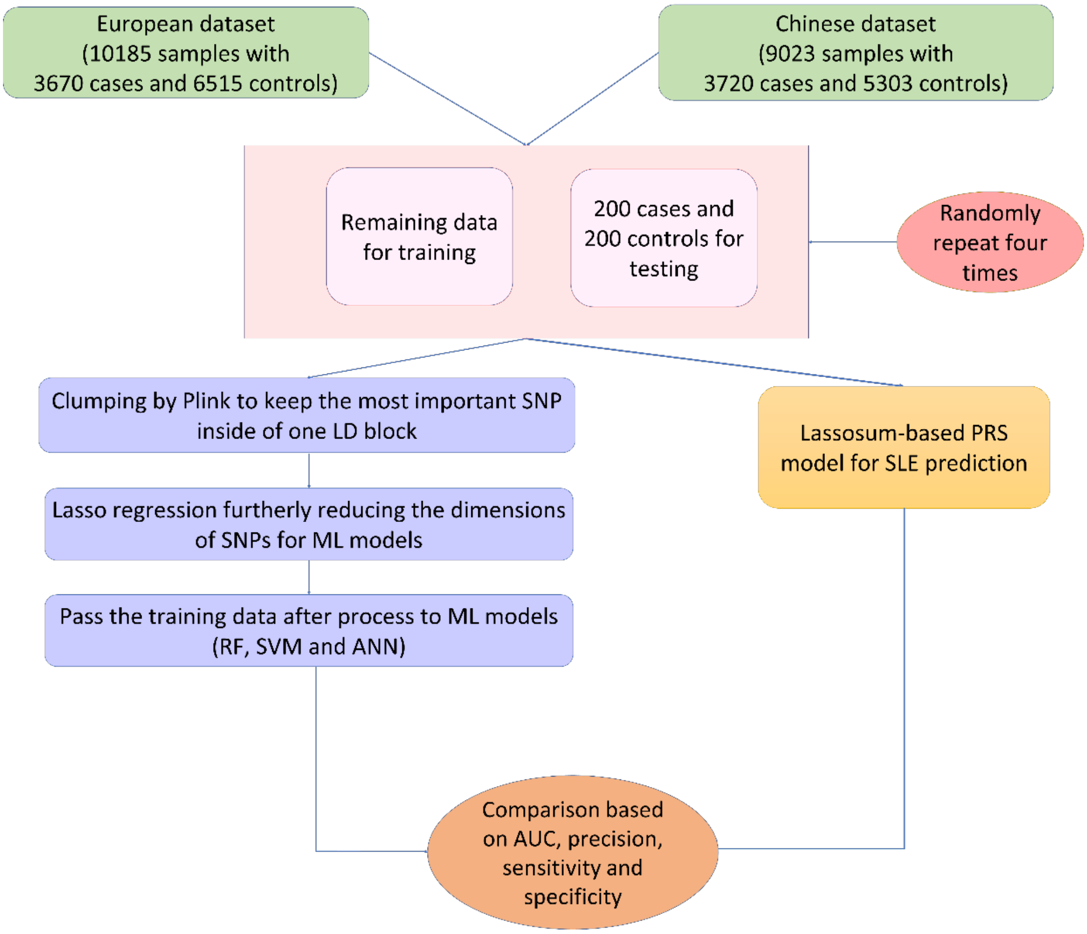

Supplementary figure 2 An overview of workflow for the internal validation of predictive models based on the data from European and Chinese datasets.

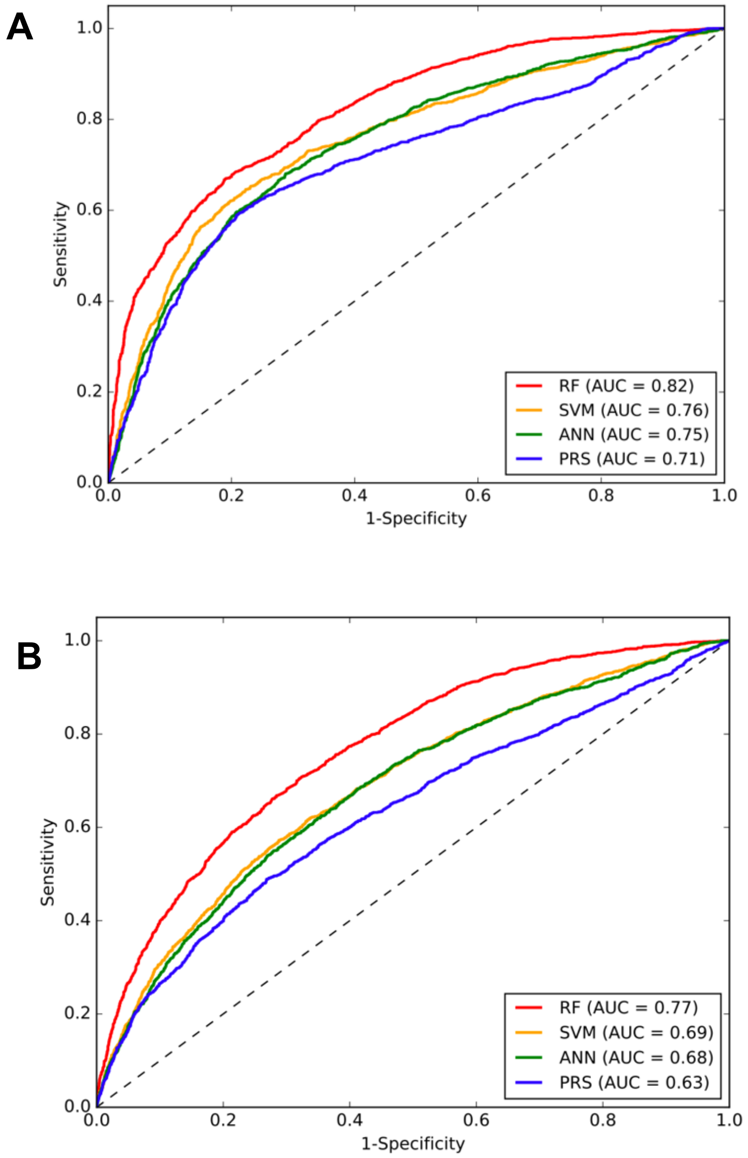

Supplementary figure 3 Performance of the random forest (RF), support vector machine (SVM), artificial neural network (ANN) and the lassosum-based PRS models for SLE prediction using either Chinese (**A**) or European datasets (**B**). Here, we randomly split the data into two parts and used one half for training and the other half for testing in each population.

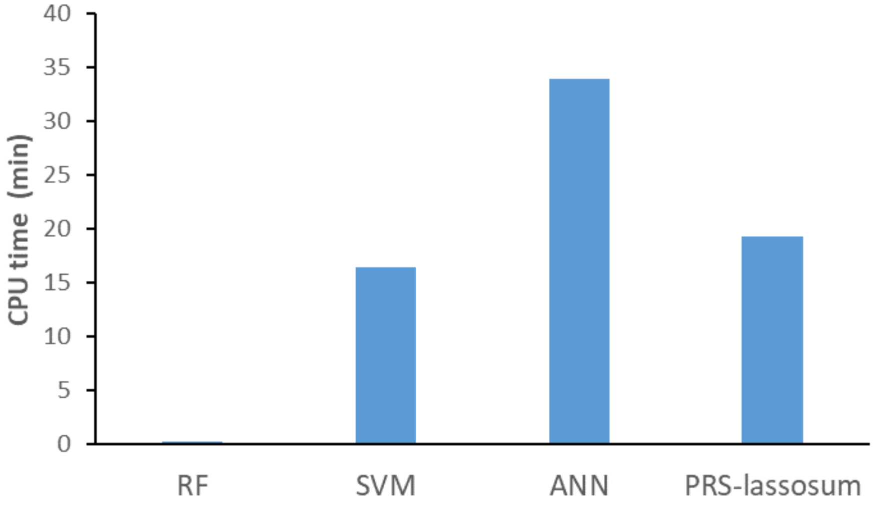

Supplementary figure 4 Average CPU time cost for the random forest (RF), support vector machine (SVM), artificial neural network (ANN) and the lassosum-based PRS models in predicting SLE development using the GWAS from Chinese population.

Supplementary table 1 Summary of SLE GWAS datasets in this study

Cohorts	Ancestry	#Case	#Controls	#Total	PMID #
HK GWAS	CHN	1,604	3,324	4,928	33536424
GZ GWAS	CHN	1,604	985	2,589	33536424
JN GWAS	CHN	512	994	1,506	33493351
EUR GWAS1	EUR	910	430	1,340	27399966/33536424
EUR GWAS2	EUR	2,354	5,379	7,733	27399966/33536424
EUR GWAS3	EUR	406	706	1,112	27399966/33536424

Supplementary table 2 setting of hyperparameters for the ANN model

Hyperparameters	Values
Batch Size	32
No. of epochs	200
No. of Hidden Layers	3
No. of Nodes in each Hidden Layer	[1024, 512, 256]
Learning Rate	0.01
Momentum	0.9
Drop-out Rate	0.3
Activation Function for each layer	‘relu’
Optimizer	Stochastic gradient descent
Strategies to avoid over-fitting	Early stopping with 10-fold CV
